# Optimised Chirped Fibre Bragg Gratings for Detonation Velocity Measurements

**DOI:** 10.3390/s19153333

**Published:** 2019-07-29

**Authors:** Josh Pooley, Ed Price, James W. Ferguson, Morten Ibsen

**Affiliations:** 1 Optoelectronics Research Centre, University of Southampton, Southampton SO17 1BJ, UK; 2 AWE Aldermaston, Reading, Berkshire RG7 4PR, UK

**Keywords:** fibre sensing, fibre Bragg gratings, chirped gratings, detonation, velocity sensing

## Abstract

Over the last decade, the use of chirped fibre Bragg gratings (CFBGs) in detonation velocity experiments has been steadily increasing. In this paper, we show how CFBG design parameters—chirp-rate, reflectivity and apodisation—affect linearity in detonation velocity tests. It is found that the optimal CFBG detonation velocity probe should have a high chirp-rate, a low reflectivity and no apodisation. As a further demonstration of these findings, we measure detonation velocity with a 24 cm optimised CFBG; the longest CFBG test of this kind so far.

## 1. Introduction

Detonation velocity is the propagation speed of a shockwave that is continuously driven through an explosive medium via exothermic chemical reactions [1]. For researchers concerned with the characterisation and assessment of explosive materials, detonation velocity can be a particularly elucidative parameter [2].

In order to measure detonation velocity accurately, methods should be as non-invasive as possible, with fast read-out speeds and reliable calibration. Throughout many decades, several detonation velocity measurement techniques have been demonstrated. Long established techniques include ionisation or piezoelectric timing pins [1], streak and framing cameras [1], and continuous resistance electrical probes [3]. More recent methods, often employing optical fibres, include fibre-coupled light detection [4], drilled fibre probes [5] and aluminium coated fibres (as timing pins) [6].

In recent years, embedded chirped fibre Bragg gratings (CFBG) have attracted significant attention in the industry [7,8,9,10]. Due to their small size, simple setup and continuous measurement capabilities, they are a convenient alternative to many of the aforementioned techniques, which are discrete, too bulky to embed, not immune to electromagnetic interference or require a direct optical sight-line.

Whilst the CFBG method of measuring detonation velocity has been successfully demonstrated a number of times, it has not yet undergone optimization to determine the optimal CFBG design parameters. In particular, spurious effects towards the end of the grating erasure can sometimes arise which are not representative of the behaviour of the detonation front and can be minimised through appropriate grating design.

In this paper, we discuss and demonstrate the effects of CFBG bandwidth and reflectivity on measurement linearity during detonation. It is shown through simulations and explosive tests that high bandwidth and low reflectivity CFBGs are less susceptible to these spurious non-linear end effects. We also show, through simulation, that CFBG apodisation is unlikely to be beneficial for detonation velocity measurements.

As a further demonstration of these findings, we measure detonation velocity with a 24 cm optimised CFBG; the longest CFBG test of this kind so far.

## 2. CFBG Concept and Simulation

### 2.1. Basic Concept

A chirped fibre Bragg grating (CFBG) is a highly dispersive optical filtering device, where stop-band wavelength components are spatially distributed along a length of optical fibre. This is achieved via inscription of a periodic refractive index change in the core of the fibre, where the periodicity is monotonically increased or decreased along the fibre length [11].

**Figure 1:**
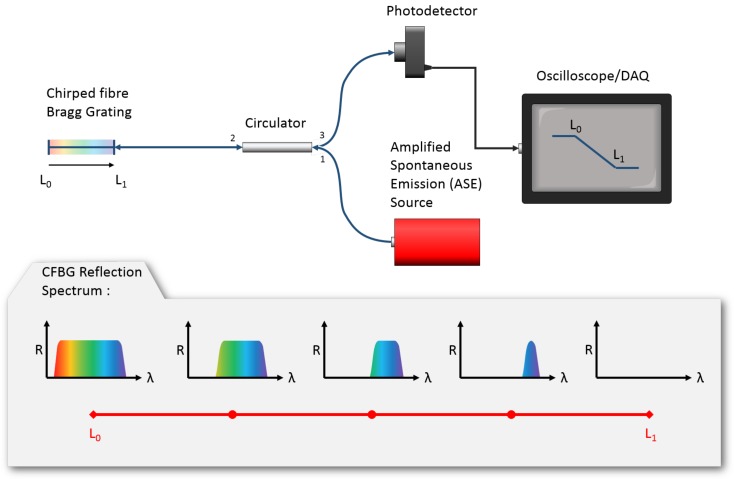
Schematic showing the interrogation setup for CFBG velocity probes. A broad bandwidth ASE source illuminates a chirped grating. The reflected signal-power is monitored on a photodetector.

Since the CFBG bandwidth and length are coupled by the *chirp-rate* of the grating, any event which results in the physical destruction of a part of the CFBG will also result in a proportional drop in the reflection bandwidth. This change in bandwidth can be monitored as a change in the total reflected power, using the setup shown in Figure 1.

In this case, a CFBG is illuminated with a broadband signal, such as that from an amplified spontaneous emission (ASE) source, which exceeds the bandwidth of the grating. The reflected light from within the CFBG bandwidth is then collected using a circulator and measured on a photodetector.

Due to the significant peak-pressures involved, if a CFBG were mounted on/in a detonating explosive device, the traversing detonation-front would progressively consume it and its reflection bandwidth would decrease accordingly. By monitoring the total reflected power during detonation, with a sufficiently fast detector and oscilloscope, a continuous detonation-front position vs. time can be recorded and used to infer the detonation velocity [7,8,9,10].

In an ideal case, the change in reflected power would be linearly proportional to the CFBG length, i.e., when the power has dropped by half, the shock-front is halfway through the grating. This is rarely the case for real measurements—primarily due to spectral variations in the source power and CFBG reflectivity—but also because of intrinsic non-linearities owed to the specific design of the grating. Simulations demonstrating these intrinsic non-linearities are shown in the following section.

### 2.2. Bandwidth Non-Linearity

Whilst a particular CFBG may have a linearly increasing resonance wavelength, it is not necessarily the case that this linear chirp-rate corresponds to a linearity between length and bandwidth. On this basis, when the grating has been cut-down to its terminal grating plane, the reflected bandwidth would become extremely narrow. This contradicts the behaviour of un-chirped, uniform fibre Bragg gratings (UFBGs), whose terminal grating plane will offer very little in the way of wavelength selectivity and be extremely broadband.

**Figure 2:**
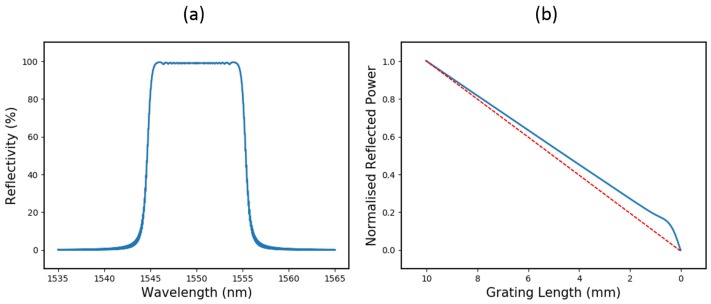
(**a**) The simulated reflection spectrum of a 10 mm, 99% reflectivity, 10 nm bandwidth, unapodised chirped grating; and (**b**) how the total reflected power reduces as the grating is destroyed. The power scales linearly until the final ~1.5 mm, where it becomes non-linear. The dashed red line shows the ideal case, where reflected power is linearly proportional to grating length.

A simulation in Figure 2 shows how the reflected power scales with length for a 10 mm, 99% reflectivity, 10 nm bandwidth, unapodised CFBG. This simulation was carried out using the transfer-matrix (T-matrix) method [12,13], where the CFBG spectrum was constructed, one sub-grating at a time, and integrated to give an accurate reflected power vs. length relationship. It can be seen that, when the CFBG reaches a certain length (~1.5 mm), the reflected power begins to scale non-linearly. This is accompanied by an 8.7% offset in the gradient when compared to the ideal linear case.

The non-linearity shown here occurs when the CFBG bandwidth approaches the bandwidth of a UFBG with the same refractive index modulation. At this point, the CFBG ceases to behave as a chirped grating and transitions into the regime of a uniform grating. The corresponding gradient deflection occurs due to depletion of the signal power reaching beyond the uniform grating regime at the launch end.

This is a relatively extreme case, but the effect it demonstrates could become a limiting factor where detonation velocity measurements are required to a high degree of accuracy. Whilst a simple two-point measurement of average detonation velocity could still be inferred from this data, as long as the start and end can be identified accurately (akin to the red dashed line in Figure 2b), non-steady-state regimes would require this gradient offset and nonlinearity to be decoupled. In order for the CFBG reflected power to scale as linearly as possible, there are two important properties which require optimisation:

- The length over which this non-linearity occurs (primarily affected by CFBG bandwidth)
- The degree of non-linearity over this region, equivalent to the gradient deflection (primarily affected by refractive index modulation i.e., CFBG reflectivity)

For an unapodised uniform grating with an effective refractive index change of δn, the full bandwidth is given by:

(1) $$\Delta \lambda_{uniform}=\frac{\lambda_{B}^{2}}{\pi n_{eff}}\sqrt{{\left(\frac{\pi \delta n}{\lambda_{B}}\right)}^{2}+{\left(\frac{\pi }{L}\right)}^{2}}$$

where $\lambda_{B}$ is the Bragg wavelength, $n_{eff}$ is the effective index of the fibre and *L* is the grating length [14].

For a chirped grating with constant chirp-rate (CR), the bandwidth is approximated as:

(2) $$\Delta \lambda_{chirped}=L\times CR.$$

**Figure 3:**
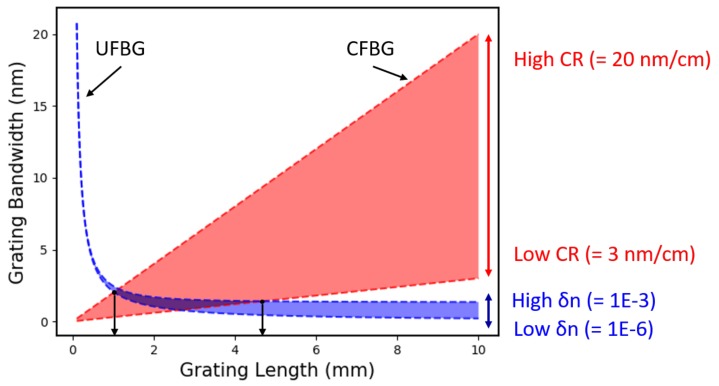
In blue: A plot of uniform grating bandwidth as a function of grating length over a range of index modulations. In red: S plot of chirped grating bandwidth over a range of chirp rates. For a CFBG with a specific chirp-rate and index modulation, the start of the non-linear region corresponds to the intersection of these two plots, where the CFBG transitions into a uniform grating.

By plotting these formulas as a function of grating length, it is possible to see the point at which a chirped grating will begin to behave as a uniform grating. Figure 3 shows the length–bandwidth relationship of UFBGs and CFBGs over a range of chirp-rates and index modulations. The intersecting region shows possible solutions, where the CFBG bandwidth is equal to the UFBG bandwidth and hence the length at which the CFBG reflected power will begin to scale non-linearly with length.

For clarity, this analysis is only shown over a 10 mm length range. However, it should be noted that these bandwidth characteristics are general for any CFBG length since it is the chirp-rate and index modulation that are the primary determinants.

It is seen here that the longest non-linear region (~4.7 mm on this plot) corresponds to a low chirp-rate and a high index-modulation CFBG. In contrast, the shortest non-linear region (~1.1 mm in this plot) corresponds to a high chirp-rate, low index-modulation CFBG. Therefore, for a chirped grating of a given length, the uniform grating region is minimised by choosing a *low-reflectivity*, *high-bandwidth* design.

Once the CFBG is in the uniform bandwidth regime, the reflected power scales non-linearly with length because the signal power is not being evenly distributed throughout the grating. For uniform gratings with a very high refractive index modulation, the penetration depth is short and only a small fraction of signal light is able to interact with the back-end. However, a uniform FBG with a very low refractive index modulation will distribute an almost equal share of signal light across the entire grating length.

**Figure 4:**
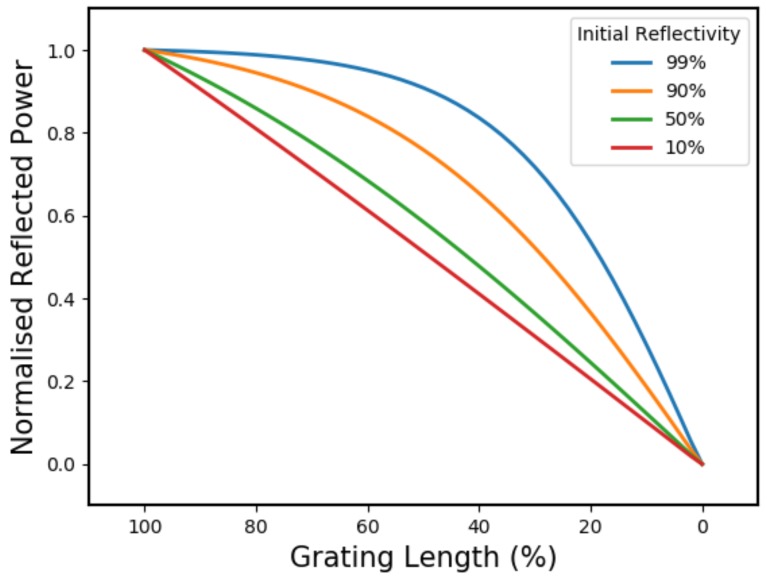
Graph showing how the total reflected power of a uniform grating (unapodised) depends on grating length and initial reflectivity. This is equivalent to an unapodised CFBG once it’s in the uniform grating regime.

Figure 4 shows a simulated plot of reflected power vs. grating length for a range of UFBG reflectivities. This is indicative of the CFBG reflected power over the non-linear end region. It can be seen that for low-reflectivity uniform gratings the power scales more linearly than for high-reflectivity gratings, where the reflected power distribution becomes increasingly front-loaded.

This signal depletion mechanism is also responsible for the total gradient offset seen in Figure 2. Since a disproportionate amount of signal light is reflected from the uniform grating region, power contributions from the rest of the grating are reduced. This means that, when half the grating has been consumed, the total reflected power will still be greater than half the initial power.

Clearly, for any CFBG, the extent of non-linearity and gradient offset is minimised with a *low-reflectivity* design.

**Figure 5:**
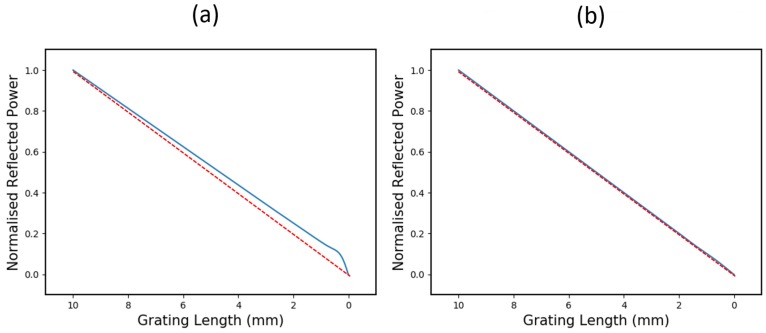
(**a**) A plot of the characteristic reflected power scaling for a 10 mm long, 20 nm bandwidth, 99% reflectivity CFBG. (**b**) A plot of the characteristic reflected power scaling for a 10 mm long, 10 nm bandwidth, 20% reflectivity CFBG.

To demonstrate the impact of reflectivity and chirp-rate on erasure linearity, the simulation shown in Figure 2 was repeated for two different CFBG designs. One, plotted in Figure 5a, shows a 10 mm long, 99% reflectivity, 20 nm bandwidth CFBG. The other, plotted in Figure 5b, shows a 10 mm long, 20% reflectivity, 10 nm bandwidth CFBG.

It is apparent, especially in the case of the low-reflectivity CFBG, that the non-linear region is drastically reduced in comparison to Figure 2, which shows a 10 mm long, 99% reflectivity, 10 nm bandwidth grating. As a quantitative measure of the reduced non-linear effect, a gradient offset has been measured for all three simulations and can be compared. In this case, the high-reflectivity, low chirp-rate CFBG in Figure 2 shows a gradient offset of 8.7%, the higher chirp-rate grating in Figure 5a shows a gradient offset of approximately 6.2% and the low-reflectivity CFBG in Figure 5b shows a gradient offset of less than 0.1%.

**Figure 6:**
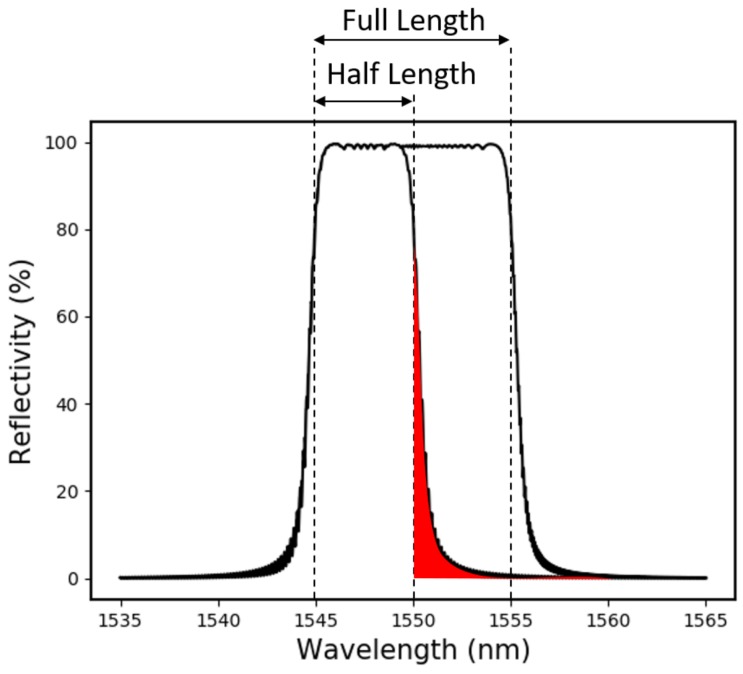
Simulation showing a high reflectivity CFBG spectrum (10 nm bandwidth, 99% reflectivity) when the grating is fully intact and when it has been cut in half. The section in red highlights the asymmetry which causes the reflected power to be front-loaded. This slight billowing out of the spectrum must also be considered when techniques are used that determine a gratings length from its bandwidth (directly).

These design considerations are also pertinent to the more recent implementations of the CFBG system, which use dispersion techniques to resolve the CFBG spectrum at high speed [15]. In this instance, it should be recognised that, particularly for high reflectivity CFBGs, the percentage bandwidth change is not directly equivalent to the percentage length change of the grating. Instead, limitations on the minimum bandwidth of a coherent section of the grating (Equation (1)) cause a billowing out of the reflection spectrum at either end. This is illustrated in Figure 6.

### 2.3. Apodisation

Many applications of CFBGs require low group-delay-ripple characteristics. This is often achieved by tapering the refractive index modulation at either end of the grating, known as apodisation [16]. The ubiquity of apodisation, especially in the telecoms sphere, means that CFBG suppliers may apodise their gratings by default. For this reason, the effects of apodisation for detonation velocity sensing are worth considering.

**Figure 7:**
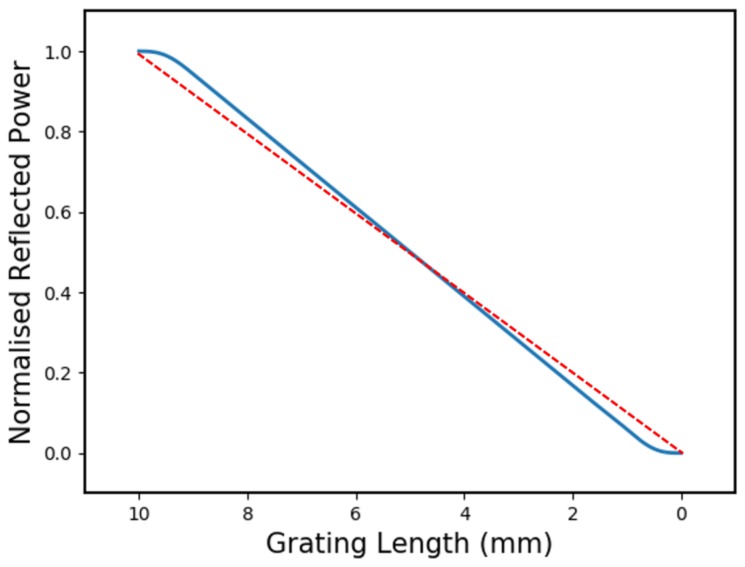
Graph showing how apodisation affects CFBGs in detonation velocity tests. The grating is 10 mm long, 20% reflectivity, with a 10 nm bandwidth and a 10% sine-shaped apodisation at either end. The apodisation can be seen as roll-in/roll-off in power as each end of the grating is destroyed. The dashed red line shows the ideal case, where reflected power is linearly proportional to grating length.

Figure 7 shows a simulation of how the reflected power scales with grating length for a 10 mm long, 10 nm bandwidth, 20% reflectivity CFBG with a 10% sine-apodisation at each end. This low-reflectivity design was chosen so that the effects of apodisation could be isolated from any bandwidth non-linearities.

In this plot, the effect of the sine-apodisation is seen as a roll-in/roll-off of the reflected power as the grating ends are consumed. This is because the tapered refractive index modulation at the each end of the grating contributes proportionally less to the total reflected power. If these end-effects are left unaccounted for, then the predicted detonation velocity could be erroneous.

This can be seen in Figure 7 by comparing the solid blue line from the apodised CFBG, with the dashed red line which connects the *true* start and end points of the grating. The difference in gradient of the two lines demonstrates how the loss of information at the apodised ends can lead to a misinterpretation of the event duration and yield a velocity which is higher than the real value.

Furthermore, any potentially favourable spectral characteristics are lost when the first apodised end is destroyed and the group-delay-ripples reappear. For this reason, apodisation is unnecessary for CFBGs in detonation velocity measurements and should be avoided.

## 3. Experimental Procedure

### 3.1. Setup

In order to demonstrate optimal CFBG parameters for detonation velocity measurements, an explosive trial was set up.

In these tests, various CFBGs were immersed in a liquid explosive—sensitised nitromethane—which was contained in a 31.75 mm outside-diameter copper cylinder and initiated from underneath via a plastic-explosive booster. The height of the cylinder was ~300 mm and the wall thickness was 3.18 mm.

**Figure 8:**
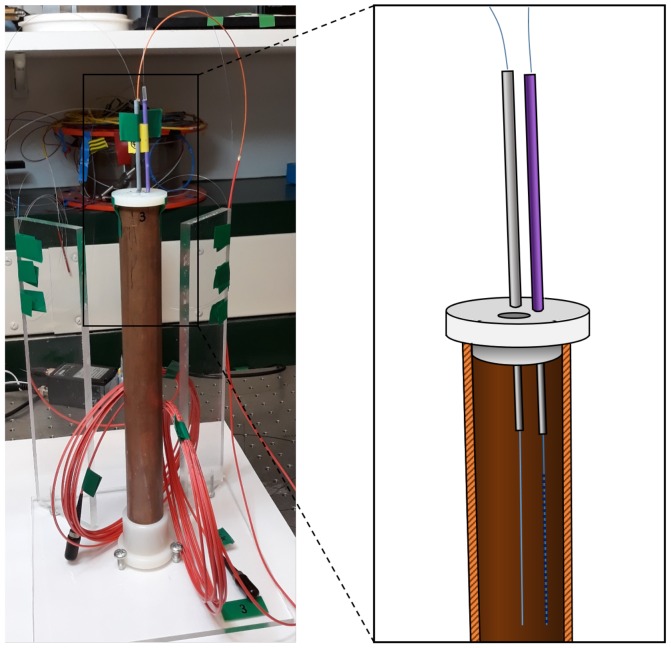
Photograph and sectional drawing of the test setup.

A nylon lid at the top of the cylinder was drilled to accommodate a number of CFBGs, which had each been spliced onto a sacrificial length of fibre. Inside the copper cylinder, the CFBGs were hanging freely and were fully exposed to the nitromethane. To help the gratings hang straight (to within an estimated uncertainty of $\pm 2^{{}^{\circ}}$), the polymer coating was removed prior to insertion into the cylinder—this drastically reduces the tendency of the fibres to bend. A photograph of the setup is shown in Figure 8.

These experiments were designed to test the CFBG probes in an ideal environment:

- Choosing a liquid explosive allowed the probes to be easily embedded without invasive adhesion techniques.
- Nitromethane is well-characterised and is known to have detonation velocity above the acoustic velocity of fused-silica [17,18].
- CFBGs were mounted to hang in the top half of the cylinder—a region which is expected to maintain a steady-state detonation (constant detonation velocity).

All of these factors combined to make a confirmatory test, where the detonation velocity can be compared against expected values and any intrinsic non-linearities from the CFBG design would be evident.

To provide a comparative velocity measurement, each CFBG test was done in conjunction with a photonic doppler velocimetry test (PDV) [19]. PDV is a well-established measurement technique which is typically used to measure surface velocities, but can be used to measure detonation velocity in this idealised scenario [20].

### 3.2. Equipment and CFBG Details

The interrogation system used to measure the CFBG reflected power during detonation is shown in Figure 1. In this case, a non-gain-flattened ASE source was used to illuminate the CFBG, with the reflected light collected on a 150 MHz InGaAs Thorlabs PDA10CF detector.

The voltage from the detector was monitored using a 10-bit National Instruments PXIe-5160 DAQ (Data Acquisition) card, which was set to a sample rate of 1.25 GSamples/s. Many of the data were subsequently low-pass filtered with a 20 MHz cutoff frequency. This was done to remove unwanted high-frequency noise and still observe non-linearities that occur over sub-mm length scales.

A piezo-electric disk was fixed to the copper cylinder and provided a trigger impulse for the DAQ card. When necessary, an optical attenuator was used prior to the grating to keep the initial reflected power below the detector saturation.

**Figure 9:**
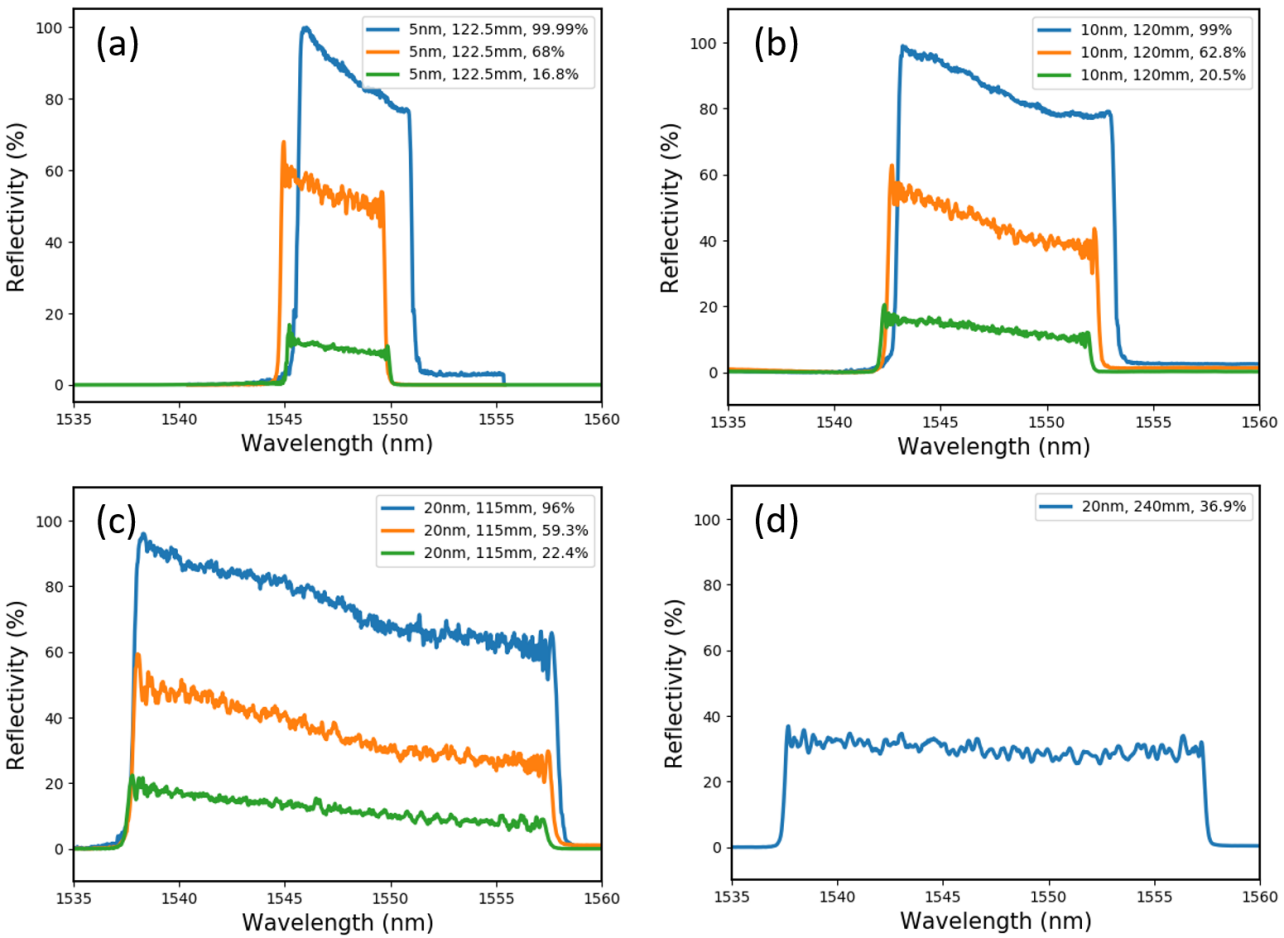
Grating spectra of the: (**a**) 5 nm bandwidth; (**b**) 10 nm bandwidth; (**c**) 20 nm bandwidth; and (**d**) 240 mm long CFBGs used in our detonation velocity tests.

Nine CFBGs were tested in nine separate experiments. Each CFBG was designed to have a 5 nm, 10 nm or 20 nm bandwidth and a <40%, 60–70% or >95% peak reflectivity. All gratings were ~120 mm long. The spectrum of each CFBG is shown in Figure 9, along with the design specification of each.

As a final demonstration of the optimised CFBG, a 240 mm, 37% reflectivity, 20 nm bandwidth grating, was tested. The long length of the grating meant that the detonation velocity could be measured throughout the majority of the event, with the CFBG hanging just ~10 mm above the booster.

All of these gratings were fabricated using a continuous writing technique, which employs a 1-m-range translation stage and interferometric position monitoring to achieve a high spatial precision [21].

## 4. Results and Discussion

**Figure 10:**
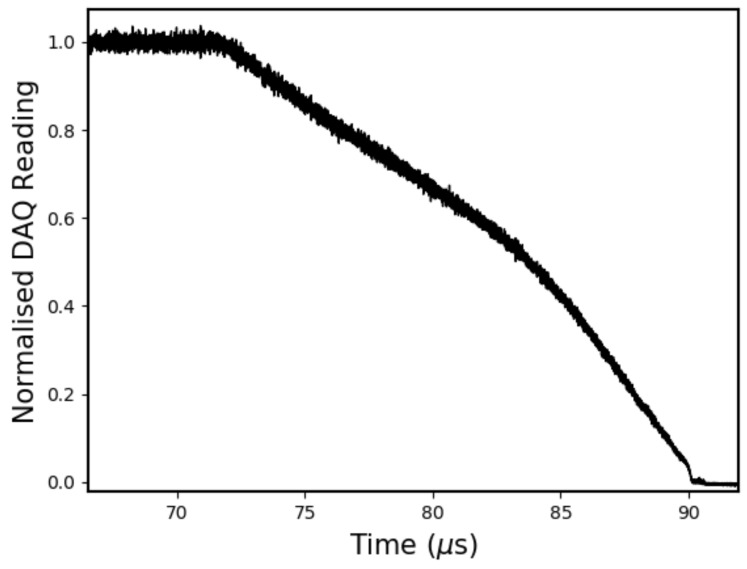
Normalised raw data from 20 nm bandwidth, 96% reflectivity, 115 mm CFBG test.

The raw data from a 20 nm bandwidth, 96% reflectivity, 115 mm CFBG test are shown in Figure 10. In this plot, which is typical of all the CFBG test data, the presence of optical signal noise is clear. At the start of recording, when the grating is fully intact, the standard deviation of this noise is ~1% of the full signal range. It is our speculation that this noise is the result of an incoherent ASE source interacting with a structure which is prone to phase errors and time-delay fluctuations [22].

**Figure 11:**
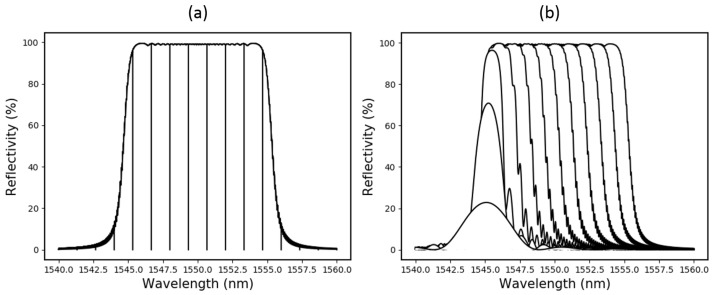
(**a**) How a reflection CFBG spectrum can be integrated, with the bandwidth incrementally removed, to create a crude calibration plot; and (**b**) a more accurate calibration plot, where the spectrum is integrated as the length of a simulated CFBG is decreased.

Since the ASE source was not gain-flattened, the raw data are S-shaped in correspondence with the variations in the power spectral density (PSD). To calibrate these data, the ASE illuminated CFBG reflection spectrum was integrated as sections of the bandwidth were digitally removed. A simulation of this process is shown in Figure 11a. For a CFBG with a known chirp-rate, this calibration data can be compared with the experimental data to get a distance vs. time plot of the detonation shock-front.

Based on the discussions in Section 2.2, it is clear that this is a crude calibration technique. For a more accurate calibration curve, the spectrum of the grating should be reconstructed in a simulation and then integrated as chunks of the simulated grating (not bandwidth) are removed. This concept is demonstrated in Figure 11b.

However, the reconstruction of a grating based on a reflection spectrum is not straight forward—instead, we intend to keep the former, cruder calibration method and show that it is a reasonable approximation for the optimised CFBG design cases demonstrated here.

**Figure 12:**
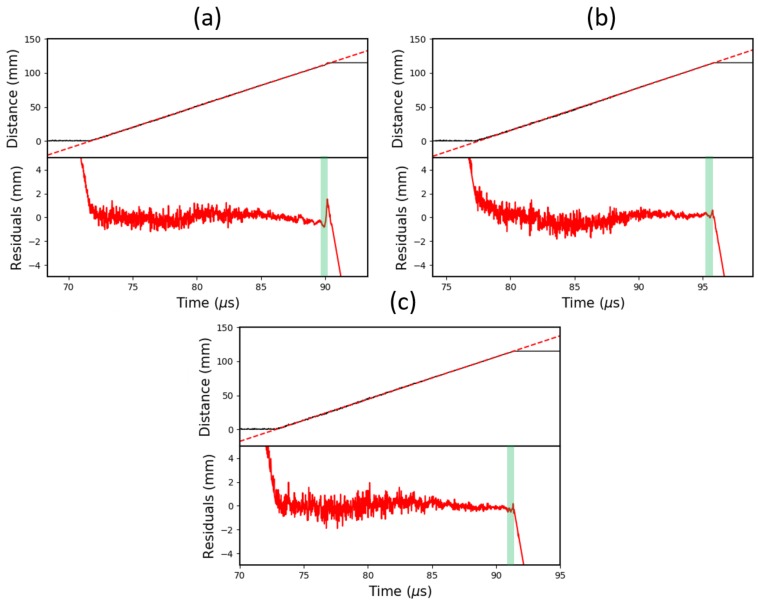
Graphs showing the calibrated and low-pass filtered (20 MHz) data from three 20 nm CFBG tests with: (**a**) 96% reflectivity; (**b**) 59% reflectivity; and (**c**) 22% reflectivity. Each CFBG is approximately 120 mm long. Plotted underneath are the residuals from the linear regression. The non-linear region, highlighted in green, is most prominent in the 96% reflectivity CFBG.

Figure 12 shows the calibrated data from three 20 nm CFBG tests with a range of reflectivities. Each dataset has undergone a linear regression fit, with the residuals plotted underneath and the non-linear region highlighted. To better visualise the underlying response of the grating, a 20 MHz low-pass filter was also applied.

Accompanying the plots is Table 1, which shows a numerical comparison of each CFBG based on analysis of the residuals. The “*Fluctuation over Highlighted Region*” column shows the peak-to-peak height of the length residuals over the non-linear region (relative to the total length). This is equivalent to the extent of gradient offset in the original data. The “Relative Length of Highlighted Region” column shows the percentage of the end of the grating that appears to be operating in the uniform grating regime.

Based on the power depletion argument in Section 2.2, the extent of the gradient fluctuation was anticipated to be greater for higher reflectivity gratings. This trend is noticeable in Table 1, where a 2.01±0.04% fluctuation in the 96% reflectivity grating drops down to just 0.48±0.09% in the 59% reflectivity grating. Beyond this, there is no measurable difference between the 22% and 59% reflectivity CFBGs, which is likely to be a result of the fluctuations becoming comparable in magnitude to the total noise levels.

In Figure 3, it is clear that the length of the CFBG non-linear region is relatively insensitive to changes in the effective index (reflectivity). This is reflected in Table 1, which shows a modest decline of 0.87±0.63% from 96% to 22% reflectivity.

The average velocities, gathered from each linear regression of the data, also agree well with the velocities obtained from the PDV results. The 96% reflectivity grating appears to yield the least accurate results, showing a velocity discrepancy of 1.6% (below the PDV result), whereas the 22% reflectivity grating is within 0.01%, measuring a velocity of 6.212 km/s (to 3 d.p.).

**Figure 13:**
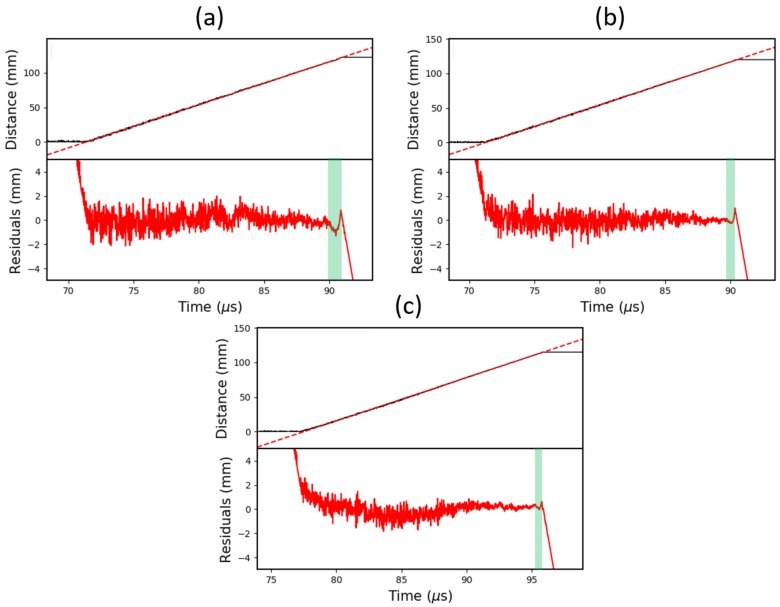
Graphs showing the calibrated and low-pass filtered (20 MHz) data from three 60–68% reflectivity CFBG tests with: (**a**) 5 nm bandwidth; (**b**) 10 nm bandwidth; and (**c**) 20 nm bandwidth. Each CFBG is approximately 120 mm long. Plotted underneath are the residuals from the linear regression. The non-linear region, highlighted in green, is longest for the 5 nm bandwidth CFBG.

A similar comparison for CFBGs with different bandwidths is shown in Figure 13 and Table 2. In this case, each grating has a reflectivity of 59–68%, and the bandwidths are 5 nm, 10 nm and 20 nm.

Due to the bandwidth argument demonstrated in Figure 3, it was expected that a higher bandwidth (higher chirp-rate) CFBG would exhibit non-linear behaviour over a shorter distance. This concept is reinforced by the data in Table 2, which show a decline in the non-linear length by more than a factor of two from 5 to 20 nm bandwidths.

Interestingly, there is also a noticeable decrease in the fluctuation amplitude from 1.44% to 0.48% for broader bandwidths. This is because the power depletion effect scales proportionally as the uniform grating region becomes enlarged i.e., for narrower bandwidths. This results in an increased *absolute* residual fluctuation.

When the average velocity data is compared to the PDV results for each of the various bandwidth CFBGs, there is little to tell them apart. All three gratings measured a velocity that was within 0.35% of the PDV results—a total average of 6.238 km/s.

**Figure 14:**
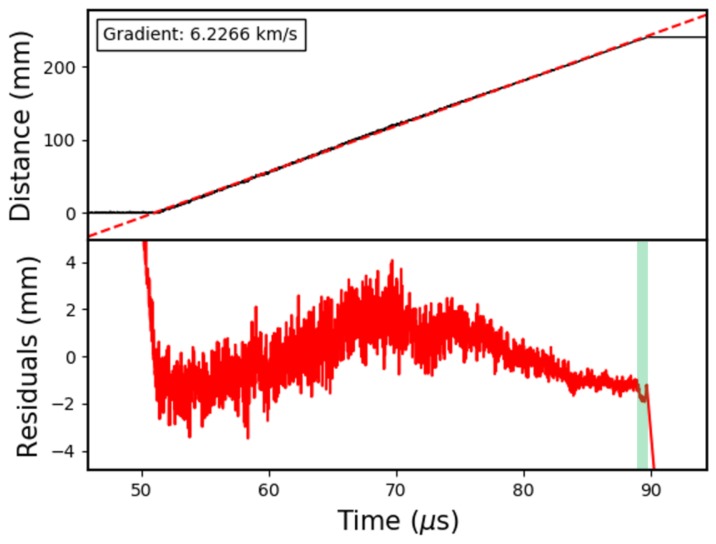
Plot showing the data from a 24 cm, 20 nm bandwidth, 37% reflectivity CFBG test.

As a final demonstration of this system, a 240 mm CFBG, was tested, which had a 20 nm bandwidth and a 37% reflectivity. This design was chosen to further demonstrate an optimised CFBG—with low-reflectivity and broad-bandwidth—as well as to demonstrate the scalability of the CFBG technique to longer lengths. The results are shown in Figure 14, with the numerical analysis shown in Table 3.

The fluctuation of 0.26±0.04% is consistent with the other low-reflectivity CFBGs shown in Figure 12 and Table 1, but is a factor of two smaller due to the approximately double-length grating. It should also be noticed that the relative length of the non-linear region is half that of the previously tested 10 nm bandwidth grating. This is because the 240 mm long, 20 nm bandwidth grating has an identical chirp-rate to the 120 mm long, 10 nm bandwidth CFBG. It should be expected, therefore, that the *relative* non-linear length in the 240 mm case, 1.60 ± 0.12%, is half as long as the 120 mm case, 3.22 ± 0.24%.

### CFBGs for Detonation Velocity Measurements: Optimal Design Summary

It is established in the previous sections that. for increased linearity, CFBG detonation velocity probes should be designed to have a low-reflectivity and broad-bandwidth (high chirp-rate).

Based on the system setup shown here, a good reflectivity range is around 20–60%. The chirp-rate of the gratings should be as high as possible, whilst considering the available source bandwidth and detector responsivity.

This design should also be relatively simple to implement from a practical standpoint. For high chirp-rate CFBGs, it can be difficult to achieve a high reflectivity because the required refractive index modulation is too great [23]. Where a low-reflectivity grating is desired, the fabrication would tend to be easier, allowing for more flexibility in the design.

Furthermore, for many of the high-reflectivity CFBG designs, an attenuator was required in order to prevent detector saturation. When the grating reflectivity was dropped, the attenuator was no longer required and the setup was simpler.

It is also shown in Figure 7 that apodisation will de-linearise the ends of the grating erasure, making calibration more difficult without any discernible benefits.

For certain detonation velocity measurements, researchers may decide to apply a second CFBG filter prior to the detector with the aim of removing out-of-band Fresnel reflections and coupled explosive light. From the results shown here, it appears that the interaction of the ASE source with the CFBG is contributing to the optical signal noise.

It is, therefore, our speculation that any additional CFBG filtering is likely to contaminate the signal with an additional background noise-level. However, in experiments where there may be considerable explosive radiation coupled into the fibre, an additional filter could contribute to a net gain in the signal quality. This should be considered on a case by case basis.

Lastly, it should be noted that CFBGs generally need to be implemented with the source signal entering via the short wavelength end so that the long wavelength end is destroyed first. This is to prevent cladding-modes leaching signal power from the short wavelengths and leaving the grating spectrum lop-sided [24].

A summary of our optimised system is shown in Table 4.

## 5. Conclusions

As CFBG methods of measuring detonation velocity are becoming more wide spread, it is crucial that subtleties in the optical setup are considered in order to maximise precision and mitigate errors. This is particularly pertinent when attempting to measure dynamic effects and changes in velocity. It is demonstrated here that broad bandwidth, low-reflectivity CFBGs are optimally linear in their response to length changes and do not require apodisation.

From here on, prospective explosives engineers and researchers should be able to optimise their measurement systems for various test scenarios. For a desired measurement length, the considerations could be:

- What is the maximum chirp-rate available—how does the full bandwidth compare to the source bandwidth and detector responsivity?
- What is the detector saturation level—what CFBG reflectivity will ensure that the reflected power does not exceed it?
- How much residual explosive light is likely to couple into the fibre—will the noise be reduced or exacerbated by an additional CFBG filter?

These consideration should go someway to helping improve the quality and reliability of CFBG detonation velocity measurements in the future.

## Figures and Tables

**Table 1:** The properties of the non-linear region for different reflectivity (96%, 59%, and 22%), 20 nm bandwidth CFBG tests.

CFBG Reflectivity (%)	Fluctuation over Highlighted Region (%)	Relative Length of Highlighted Region (%)	Average Velocity (km/s)	PDV Velocity (km/s)
96	2.01 ± 0.04	2.87 ± 0.32	6.150	6.248
59	0.48 ± 0.09	2.12 ± 0.65	6.259	6.238
22	0.48 ± 0.13	2.00 ± 0.54	6.212	6.212

**Table 2:** The properties of the non-linear region for different bandwidth (5 nm, 10 nm, and 20 nm) CFBG tests, with reflectivities in the range 59–68%.

CFBG Bandwidth (nm)	Fluctuation over Highlighted Region (%)	Relative Length of Highlighted Region (%)	Average Velocity (km/s)	PDV Velocity (km/s)
5	1.44 ± 0.08	4.88 ± 0.67	6.234	6.247
10	1.03 ± 0.04	3.22 ± 0.24	6.223	6.244
20	0.48 ± 0.09	2.12 ± 0.65	6.259	6.238

**Table 3:** The properties of the non-linear region for a 24 cm, 20 nm bandwidth, 37% reflectivity CFBG test.

Fluctuation over Highlighted Region (%)	Relative Length of Highlighted Region (%)	Average Velocity (km/s)
0.26 ± 0.04	1.60 ± 0.12	6.227

**Table 4:** A summary of design considerations for CFBG detonation velocity tests.

Design Parameter	Optimal Implementation	Explanation
CFBG Chirp-Rate/Bandwidth	✓ High Chirp Rate	A high chirp-rate minimises the length of the non-linear region.
CFBG Reflectivity	✓ Low Reflectivity	A low reflectivity reduces the severity of the non-linear region and simplifies fabrication of high chirp-rate gratings.
Signal Input/Output End	✓ Short Wavelength End	Source light should enter and leave the grating from the short wavelength end to avoid cladding-mode loss.
Additional CFBGFiltering	━ Apply with Caution	Some applications may require additional filters, but in other cases the extra CFBG could contribute more noise than it filters out.
Source Attenuation	━ Can be Avoided	Attenuation can be avoided by designing CFBGs whose reflected power is below the detector saturation level.
CFBG Apodisation	✗ No Apodisation	Apodisation will tend to de-linearise the length vs. reflected power at each end of the grating.

## Abbreviations

The following abbreviations are used in this manuscript:

ASE	Amplified Spontaneous Emission
CFBG	Chirped Fibre Bragg Grating
CR	Chirp-Rate
DAQ	Data Acquisition
PDV	Photonic Doppler Velocimetry
PSD	Power Spectral Density
UFBG	Uniform Fibre Bragg Grating
